# 1600. Long-Acting Antiretroviral Injectable: Who Would Benefit?

**DOI:** 10.1093/ofid/ofad500.1435

**Published:** 2023-11-27

**Authors:** Karolina Pogorzelski, Dima Dandachi, Nicole M Hitchcock, Michela Fabricius

**Affiliations:** University of Missouri-Columbia, Columbia, Missouri; University of Missouri - Columbia, Columbia, Missouri; University of Missouri, Carthage, Missouri; University of Missouri School of Medicine, Columbia, Missouri

## Abstract

**Background:**

People with HIV (PWH) face many challenges in obtaining HIV care. Long-acting (LA)-injectable antiretroviral therapies (ART) can address some of these barriers. This project aimed to determine the percentage of patients who could receive the LA-injectable ART cabotegravir/rilpivirine (CAB/RPV) as an alternative to an oral daily pill regimen based on clinical indications.

**Methods:**

We retrospectively reviewed medical records of adult PWH seen at the University of Missouri Health Care (MUHC) HIV outpatient clinic in Columbia, MO. We collected demographics, CD4 counts, hepatitis B status, HIV viral load, and HIV genotype resistance data. The primary endpoint was the percentage of PWH who would qualify to receive the LA-injectable ART CAB/RPV based on an undetectable HIV viral load, no documented chronic hepatitis B status, and no resistance to either CAB or RPV based on the Stanford University HIV Drug Resistance Database.

**Results:**

Six hundred and twenty-five unique PWH were receiving care at MUHC with a median age of 49 years old, 20% female, 27.5% Black/African American, 4.8% Hispanic and/or Latino, and 9.1% with AIDS (CD4 count < 200 cells/uL). 17.9% of PWH were not eligible for the LA-injectable ART CAB/RPV due to a detectable HIV viral load (defined as > 50 copies/mL on the last HIV viral load result). An additional 7.2% did not qualify due to having a genotype resistance to CAB or RPV and 1.8% did not qualify due to having chronic hepatitis B. Altogether, 151 PWH (24.2%) had one or more clinical contraindications for the LA-injectable ART CAB/RPV based on current guidelines. Genotype resistance testing was not available for 59.6% of PWH. However, out of the 474 PWH who are eligible to receive the LA-injectable ART CAB/RPV, only 76 (16.1%) are currently in care and on this regimen.

**Figure:**
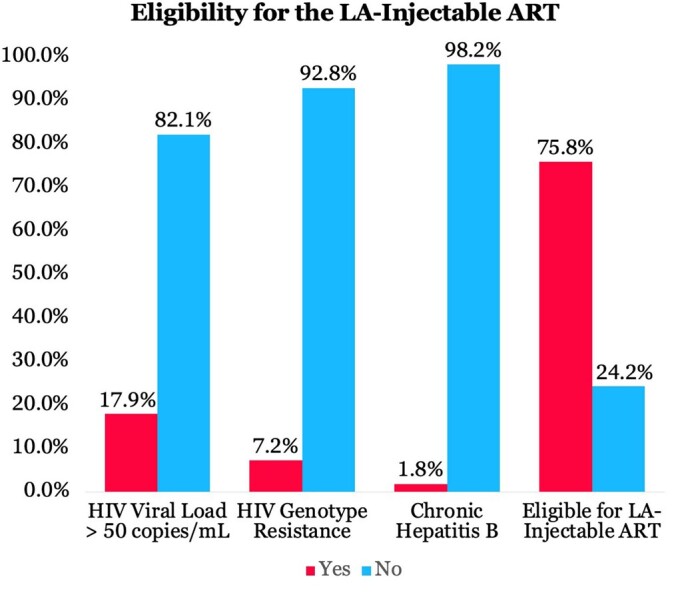

**Figure:**
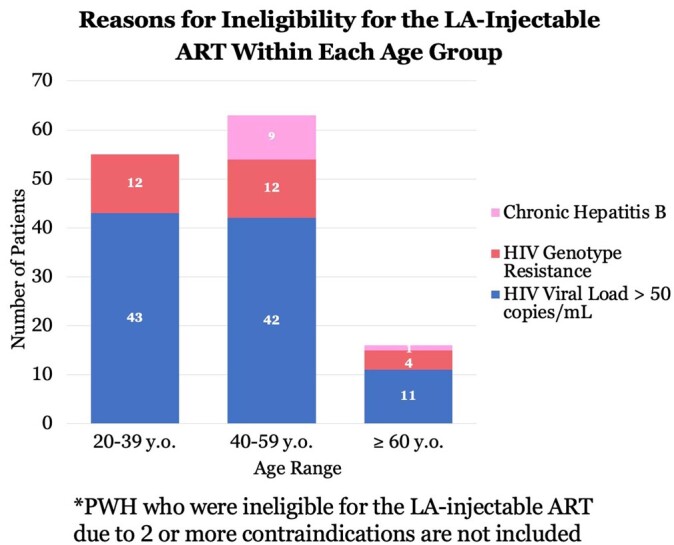

**Conclusion:**

In this study, we identified a high percentage of patients with no available genotype and a low uptake of the new LA-injectable ART CAB/RPV. To optimize the use of the the LA-injectable ART CAB/RPV and to make it accessible to PWH who could benefit from it, further research needs to be done using both quantitative and qualitative measures to identify barriers and facilitators at the patient, provider, and system level and to develop effective implementation strategies.

**Disclosures:**

**Dima Dandachi, MD, MPH**, ContraFect Corporation: Grant/Research Support|Missouri Foundation for Health: Grant/Research Support|ViiV healthcare: Grant/Research Support|ViiV healthcare: Honoraria

